# Transient Plasmacytosis With Trisomy of Chromosome 8 in a Patient With Multiple Myeloma: A Case Report

**DOI:** 10.4021/wjon688w

**Published:** 2013-09-27

**Authors:** Nobuhiro Akuzawa, Takashi Hatori, Kunihiko Imai, Yonosuke Kitahara, Shinji Sakurai, Masahiko Kurabayashi

**Affiliations:** a Department of Internal Medicine, Social Insurance Gunma Chuo General Hospital, 1-7-13 Koun-cho, Maebashi, Gunma 371-0025, Japan; b Department of Pathology, Social Insurance Gunma Chuo General Hospital, 1-7-13 Koun-cho, Maebashi, Gunma 371-0025, Japan; c Department of Medicine and Biological Science, Gunma University Graduate School of Medicine, 3-39-22 Showa-Machi, Maebashi, Gunma 371-8511, Japan

**Keywords:** Multiple myeloma, Plasmacytosis, Trisomy 8

## Abstract

A 96-year-old woman with a 5-year history of multiple myeloma was admitted to our hospital because of increasing fatigue and fever. Bone marrow plasma cell analysis showed t(11;14), del(13q), and del(17p13). Her condition deteriorated, and she developed plasmacytosis resembling plasma cell leukemia. Chromosome analysis showed trisomy of chromosome 8 in the circulating plasma cells. The plasmacytosis resolved spontaneously without chemotherapy after about 5 weeks, and the trisomy became undetectable. The findings suggest that trisomy 8 might have contributed to the transient plasmacytosis, and that chromosome 8 carries genes associated with plasma cell proliferation, maturation, and apoptosis.

## Introduction

Multiple myeloma (MM) accounts for approximately 13% of hematologic cancers. This plasma cell disorder is characterized by clonal proliferation of malignant plasma cells in the bone marrow, monoclonal proteins in the blood and urine, and associated organ dysfunction [1]. Past studies have reported relationships between specific cytogenetic abnormalities and prognosis [2, 3]. Specific translocations detected by interphase fluorescence in situ hybridization (FISH) in the immunoglobulin heavy chain region such as t(4;14), del(17p13), and chromosome 1 abnormalities are associated with poor prognosis [4]. Leukemic transformation of MM results in secondary plasma cell leukemia (PCL) in 1.8-4% of cases [5], but the molecular basis of PCL remains poorly understood.

We present here a rare case of MM that showed transient plasmacytosis resembling PCL with trisomy of chromosome 8. The plasmacytosis resolved spontaneously, after which trisomy 8 became undetectable.

## Case Report

**Figure 1:**
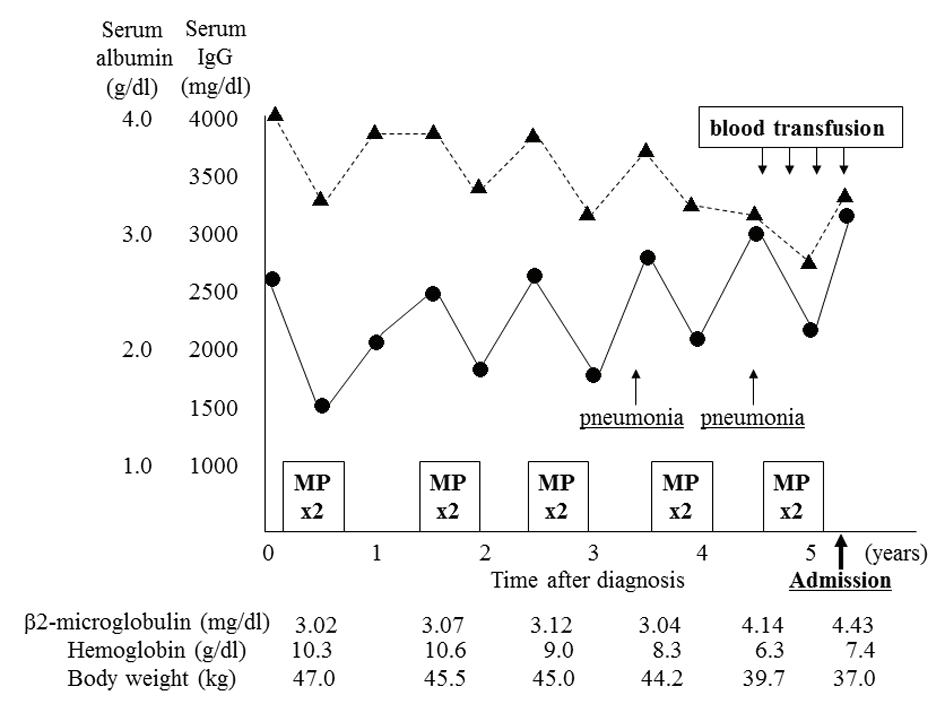
Clinical course before admission. The patient underwent five courses (two cycles each) of combination therapy with melphalan and prednisolone (MP) before admission. Although chemotherapy had been effective, serum albumin level (Alb, ▲) and hemoglobin level gradually decreased, and IgG level (•) and β2-microglobulin level increased. She was admitted twice for the treatment of bacterial pneumonia. She underwent a conspicuous weight loss during the year before admission, accompanied by a need for frequent blood transfusion.

A 96-year-old woman was admitted to our hospital because of increasing fatigue and fever. Five years previously, she had been admitted because of a compression fracture of her lumbar spine, and a skeletal survey had revealed multiple osteolytic lesions in the thoracolumbar spine. Bone marrow aspirate showed increased plasma cells accounting for 45% of nucleated cells, and laboratory testing showed monoclonal IgG and λ gammopathy. She was diagnosed with MM stage I, according to the International Staging System of the International Myeloma Working Group. Although conventional cytogenetic analysis of bone marrow cells showed no abnormalities, interphase FISH analysis identified the translocation t(11;14)(q13;q32). She underwent five courses of chemotherapy over 5 years with melphalan (0.15 mg/kg/day, day 1 - 4 for 5 weeks) and prednisolone (1.5 mg/kg/day, day 1 - 4 for 5 weeks). Each chemotherapy course was administered for two cycles. Oral alendronate had been administered as maintenance therapy. She had not developed renal dysfunction or additional fractures, but had been admitted to hospital twice with bacterial pneumonia. Her body weight had decreased from 44.2 to 37.0 kg over two years. During the preceding year, recurrent anemia had been treated with blood transfusion every 3 months (Fig. 1). Chromosomal, immunophenotypic, and interphase FISH analysis of bone marrow plasma cells had last been performed 6 months previously, and the findings were not significantly different to the initial findings at diagnosis. She had no other significant medical history, and no significant family history.

**Figure 2:**
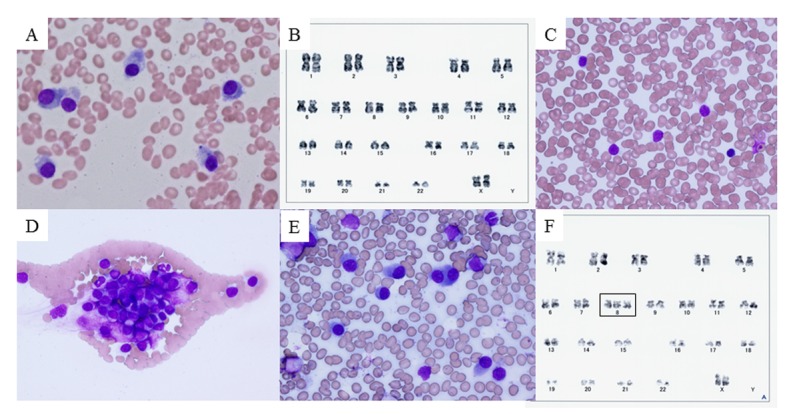
Bone marrow smear, peripheral blood smear, and chromosome analysis. (A) Bone marrow smear with Giemsa staining on day 3, showing plasma cells (myeloma cells) with basophilic cytoplasm and perinuclear halos. (B) Chromosome analysis of bone marrow cells on day 3, showing normal karyotype. (C, D) Peripheral blood smear with Giemsa staining on day 28, showing unclassifiable lymphoid cells with scant cytoplasm and cytoplasmic hairy projections. These cells tended to form conglomerates. (E) Bone marrow smear with Giemsa staining on day 28. Myeloma cells predominated (58.5%) and only a few lymphoid cells were observed (2.5%). (F) Chromosome analysis of peripheral blood on day 30, showing trisomy of chromosome 8 that had not previously been observed (rectangle).

On admission, she was alert with a pulse rate of 96 beats/min, temperature of 37.8 °C, and blood pressure of 102/70 mmHg. Chest X-ray was unremarkable, and no specific abnormalities were detected on physical examination. Laboratory testing showed anemia, elevated serum IgG, free λ chain, and serum β2-microglobulin levels; and markedly decreased serum IgA and IgM levels (Table 1). Cultures of sputum, urine, and blood did not grow pathogenic bacteria. As the cause of her fever remained unclear, sternal bone marrow aspirate was performed on day 3, and showed increased numbers of plasma cells accounting for 48.8% of nucleated cells, with no significant morphological differences compared with the previous examination (Fig. 2A). The plasma cells were positive for CD20, CD38, CD138, MPC-1, and cytoplasmic λ-chain; and negative for CD19 and CD56; which was the same as in previous examinations. Although chromosome analysis showed a normal karyotype (Fig. 2B), interphase FISH analysis showed t(11;14)(q13;q32), del(13q), and del(17p13). Her body temperature remained at about 38 °C, and her general condition rapidly deteriorated. On day 5, meropenem (1.5 g/day) was started, but her fever continued. On day 10, she was no longer able to eat regular meals. On day 15, she became bedridden and total parenteral nutrition was started. She agreed to further antibiotic treatment, but refused chemotherapy. Sputum, urine, and blood cultures were repeated. No pathogens were detected in the culture specimens, and on day 18 meropenem was discontinued and ciprofloxacin (600 mg/day) was started. Computed tomography of the neck, chest, and abdomen showed no abnormalities.

**Table 1:** Laboratory Findings on Admission

Hematology	
White Blood Cells	4,700/mm^3^
Red Blood Cells*	209 × 10^4^/mm^3^
Hemoglobin*	7.4 g/dL
Hematocrit*	23.4%
Platelets	13.2 × 10^4^/mm^3^
Differential White Blood Cell Count	
Segmented neutrophils	45.0%
Band cells, stab cells	2.0%
Metamyelocytes	2.0%
Eosinophil	4.0%
Monocytes	4.0%
Lymphocyte	43.0%
Serology	
IgG*	3,040 mg/dL
IgA*	8 mg/dL
IgM*	< 5 mg/dL
Free κ chains	3.4 mg/L
Free λ chains*	97.1 mg/L
κ to λ ratio*	0.035
Biochemistry	
Total protein	7.5 g/dL
Albumin	3.1 g/dL
AST	23 IU/L
ALT	15 IU/L
LDH	210 IU/L
ALP	292 IU/L
γ-GTP	97 IU/L
Total bilirubin	0.5 mg/dL
Blood urea nitrogen	7.6 mg/dL
Creatinine	0.66 mg/dL
Sodium	136 mEq/L
Potassium	4.5 mEq/L
Chloride	109 mEq/L
Calcium	8.6 md/dL
Phosphorus	3.1 mg/dL
Glucose	93 mg/dL
C-reactive protein*	0.50 mg/dL
β2-microglobulin*	4.43 mg/L

*These values are outside the normal range. AST, aspartate aminotoransferase; ALT, alanine aminotoransferase; LDH, lactate dehydrogenase; ALP, alkaline phosphatase; γ-GTP, γ-glutamyltransferase.

On day 28, her body temperature increased to 40.3 °C. Her white blood cell count increased to 14,300/mm^3^, including 41% (5,860/mm^3^) unclassifiable cells, and her platelet count decreased to 3.6 × 10^4^/mm^3^. Peripheral blood smear showed increased numbers of atypical lymphoid cells of variable sizes, with scant cytoplasm and cytoplasmic hairy projections (Fig. 2C), which tended to form conglomerates (Fig. 2D). Repeat bone marrow aspiration showed 58.5% plasma cells with almost the same morphology as on day 3 (Fig. 2E), and 22.5% unclassifiable lymphoid cells. Chromosome analysis of bone marrow plasma cells showed normal karyotype. On day 30, immunophenotypic and interphase FISH analysis of circulating lymphoid cells showed the same characteristics as in the bone marrow plasma cells. However, chromosome analysis of circulating lymphoid cells detected trisomy of chromosome 8, which had not been detected on any previous examinations of bone marrow cells (Fig. 2F). Based on these findings, we suspected secondary PCL as a leukemic transformation of MM.

**Figure 3:**
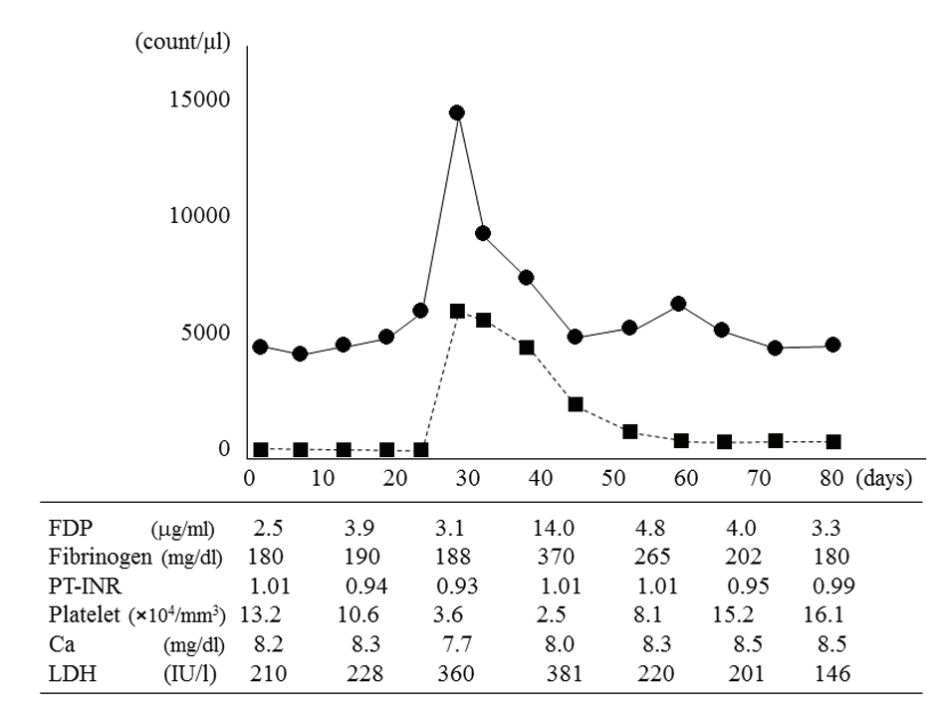
White blood cell count, atypical plasma cell count in peripheral blood, and other laboratory parameters. The white blood cell count (•) and atypical plasma cell count (■) peaked on day 28 and then spontaneously decreased. On day 42, fibrin degradation products (FDP), fibrinogen, and lactate dehydrogenase (LDH) values peaked; and the platelet count reached its minimum value. The atypical plasma cell count in peripheral blood decreased to 20/mm^3^ on day 58, but did not completely resolve. The calcium (Ca) level and prothrombin time-international normalized ratio (PT-INR) remained almost normal.

On day 42, her body temperature remained above 39 °C, and her blood platelet count decreased to 2.5 × 10^4^/mm^3^ (Fig. 3). Levels of fibrin degradation products increased to 14.0 µg/mL, fibrinogen increased to 370 mg/dL, and lactate dehydrogenase increased to 381 IU/L. Even though no chemotherapy was administered, the atypical peripheral plasma cell count decreased to 20/mm^3^ on day 58. Bone marrow aspiration on day 58 showed 62% plasma cells with undetectable lymphoid cells. Trisomy 8 was undetectable both in bone marrow and peripheral blood cells. Interphase FISH analysis of bone marrow plasma cells continued to show t(11;14)(q13;q32), del(13q), and del(17p13). On day 60, her body temperature returned to normal, and on day 72, all other laboratory parameters had returned to normal. Another bone marrow aspirate showed plasma cells accounting for 58.2% of nucleated cells, and < 0.5% atypical lymphoid plasma cells. She was eager to go home, although she continued to be fatigued and was unable to tolerate oral intake. After implantation of a subcutaneous access port in her left chest wall for intravenous nutrition, she was discharged on day 90 with medical support and home assistance, and regular follow-up by her family doctor. During the 5 months since discharge, her white blood cell count had been about 4,000/mm^3^ and her general condition had remained stable, but she died of bacterial pneumonia 6 months after discharge.

## Discussion

Our patient had markedly increased circulating lymphoid plasma cells after a month of fever. The maximum value of lymphoid plasma cells in the peripheral blood was 5,860/mm^3^, accounting for 41% of white blood cells. These values satisfied the criteria for PCL described by Kyle et al [6], including an absolute plasma cell count > 2 × 10^3^/mm^3^, with plasma cells comprising > 20% of peripheral blood cells. No pathogens were detected in any of her culture samples, and antibiotic administration did not resolve her fever. In addition, trisomy 8 was detected only in circulating lymphoid plasma cells. These findings suggest that proliferation of variant plasma cells might have contributed to her fever.

Interestingly, the plasmacytosis resolved spontaneously. The morphology of the circulating atypical plasma cells with cytoplasmic projections was similar to those of previously reported patients with primary PCL [7, 8]. Circulating plasma cells were negative for both CD45 and CD49e, and positive for MPC-1, which is the same as in intermediate myeloma cells [9]. Activation of the Src family kinases associated with CD45 expression is a prerequisite for proliferation via interleukin-6 in myeloma cells [9]. Thus, loss of CD45 expression may stop the excessive proliferation of atypical plasma cells in this patient. Interphase FISH analysis showed t(11;14)(q13;q32), del(13q), and del(17p13) in both circulating and bone marrow plasma cells, although only t(11;14)(q13;q32) was observed at 6 months before admission. The translocation t(11;14), known to occur in patients with dysregulation of cyclin D1 and myeloma overexpressed gene (MYEOV) [10], is present in 21% of MM patients [4]. This translocation is associated with increased expression of CD20, down-regulation of CD56, and small mature plasma cells [11, 12], as also observed in our patient. Some studies reported that t(11;14) and del(13q) were not associated with survival time in MM patients [4, 13], and that MM patients with t(11;14) showed less proliferation [10]. In contrast, the prognosis of primary PCL patients with t(11;14) is poor [11]. In primary PCL, the most prevalent immunoglobulin heavy chain translocation is t(11;14), occurring in 25-65% of cases, which is more common than in MM [11]. In secondary PCL, multiple translocation partners have been observed including t(11;14), t(4;14), and t(14;16) [5]. Both del(17p13) and t(4;14) are associated with poor prognosis in MM patients [1]. Coding mutations in TP53 are also common in both primary and secondary PCL [4, 11]. In our patient, a cascade of processes may have occurred before admission, with acquisition of abnormalities that are often seen in MM cases with poor prognosis and secondary PCL. The addition of trisomy 8 might then have induced plasmacytosis. In this patient, trisomy 8 was only observed in circulating lymphoid cells. In patients with MM, hyperploidy is not generally considered to affect prognosis, in contrast to hypoploidy, which is associated with poor prognosis [14]. However, the reported 1-year mortality rate of MM patients with trisomy 8 is 87% [10, 15]. Trisomy 8 is observed in 8% of MM cases, which is less than the frequency of trisomy 1 (37%), trisomy 6 (32%), trisomy 9 (52%), trisomy 11 (33%), or trisomy 15 (48%) [16]; but trisomy 8 is not observed in primary PCL patients despite a high frequency of trisomy 1 (43%) and trisomy 18 (43%) [16]. Robak et al [17]reported a rare case of primary PCL with trisomy 8. Additionally, an MM patient with trisomy 8 was reported to develop nonlymphocytic leukemia with the same karyotypic abnormality as the preexisting myeloma cells [18]. However, no cases of secondary PCL with trisomy 8 have previously been reported. Trisomy 8 is typically observed in myelodysplastic syndrome [10], and CD34 cells from patients with trisomy 8 myelodysplastic syndrome express early apoptotic markers but avoid programmed cell death by up-regulation of antiapoptotic proteins, including c-myc, assigned to band q24 of chromosome 8 [19]. The c-myc genes are not expressed in terminally differentiated normal plasma cells, and c-myc dysregulation is a late event in MM [20]. Deregulated expression of c-myc not only promotes cellular proliferation but also either induces or sensitizes cells to apoptosis [21], and stabilization of p53 is associated with c-myc-induced apoptosis [22]. Although we could not investigate the expression of the c-myc gene because of failure to establish a cell line of circulating lymphoid cells, apoptosis associated with c-myc may have contributed to the decrease in circulating lymphoid cells.

Interestingly, the proportion of lymphoid plasma cells was relatively low in the sternal bone marrow aspirate in the present case. Compared with MM, tumor cells in primary and secondary PCL have reduced expression of adhesion molecules such as neural cell adhesion molecule-1 (CD56) and leukocyte-associated antigen-1 [11]. In our patient, both MM cells and circulating atypical plasma cells lacked CD56 expression. Decreased expression of other adhesion molecules may have contributed to plasmacytosis in the peripheral blood. It is also noteworthy that the plasmacytosis observed in this patient was transient. Dissemination of tumor cells from the bone marrow may also be affected by changes in expression of chemokine receptors, and by molecular aberrations that influence bone marrow microenvironment-independent tumor growth, inhibition of apoptosis, and escape from immune surveillance [11]. The circulating plasma cells in our patient might have lacked some of the factors necessary for continued proliferation outside the bone marrow.

In conclusion, we experienced a patient with MM who developed transient plasmacytosis. This is the first report of plasmacytosis associated with trisomy of chromosome 8 in a patient with MM. Although the morphology of the circulating plasma cells was clearly different from that of the bone marrow plasma cells, the cells had common genetic abnormalities, with the exception of trisomy 8 which was only observed in circulating plasma cells. The plasma cell count temporarily satisfied the criteria for PCL, but spontaneously returned to normal after several weeks. The addition of trisomy 8 to preexisting t(11;14), del(17p13), and del(13q) might have contributed to the transient plasmacytosis. These findings suggest that some genes carried on chromosome 8 may play a key role in the proliferation, maturation, and apoptosis of plasma cells.
